# Controlling Thermoelectric Properties of Laser-Induced Graphene on Polyimide

**DOI:** 10.3390/nano14100879

**Published:** 2024-05-19

**Authors:** Cem Kincal, Nuri Solak

**Affiliations:** Department of Metallurgical and Materials Engineering, Istanbul Technical University, 34469 Istanbul, Turkey; kincal@itu.edu.tr

**Keywords:** laser-induced graphene, thermoelectrics, thermal conductivity, wearable electronics

## Abstract

In the field of wearable thermoelectric generators, graphene-based materials have attracted attention as suitable candidates due to their low material costs and tunable electronic properties. However, their high thermal conductivity poses significant challenges. Low thermal conductivity due to porous structure of the laser-induced graphene, combined with its affordability and scalability, positions it as a promising candidate for thermoelectric applications. In this study, thermoelectric properties of the laser-induced graphene (LIG) on polyimide and their dependence on structural modifications of LIG were investigated. Furthermore, it was shown that increasing the laser scribing power on polyimide results in larger graphene flakes and a higher degree of graphitization. Electrical conductivity measurements indicated an increase with increasing laser power, due to a higher degree of graphitization, which enhances charge carrier mobility. Our findings reveal that LIG exhibits p-type semiconducting behavior, characterized by a positive Seebeck coefficient. It was shown that increasing laser power increased the Seebeck coefficient and electrical conductivity simultaneously, which is attributed to a charge carrier energy filtering effect arising from structures occurred on the graphene flakes. Moreover, the porous structure of LIG contributes to its relatively low thermal conductivity, ranging between 0.6 W/m·K and 0.85 W/m·K, which enhances the thermoelectric performance of LIG. It has been observed that with increasing laser power, the figure of merit for laser-induced graphene can be enhanced by nearly 10 times, which holds promising applications for laser-induced graphene due to the tunability of its thermoelectric performance by changing laser parameters.

## 1. Introduction

In the growing field of wearable electronics, the quest for an efficient and sustainable power source has taken center stage. As a result, adopting self-powered systems that harvest energy from the human body or the environment has become a promising approach [1]. Piezoelectric nanogenerators [2], triboelectric nanogenerators [3], and solar generators [4] have been extensively researched as self-powered systems for energy supply. However, piezoelectric and triboelectric generators require continuous motion, whereas solar generators need light. These dependencies can pose challenges in practical applications where such conditions are not always present. To overcome these issues, wearable thermoelectric generators (w-TEGs) offer a promising solution by continuously converting body heat into electrical energy through the Seebeck effect [5,6,7]. The effectiveness of thermoelectric materials is measured by a figure of merit known as ZT [8].

ZT = (S^2^σ)T/к (1)

Here, S represents the Seebeck coefficient, σ stands for electrical conductivity, κ indicates thermal conductivity, and T refers to the absolute temperature.

Currently, the thermoelectric generators available in the market primarily use inorganic materials like Bi_2_Te_3_, PbTe, Sb_2_Te_3_ due to their high ZT values [9,10,11,12]. However, these materials face significant challenges, including high toxicity, considerable costs, fragility, and processing complexities [13]. As an alternative, polymers and their composites are extensively investigated. These materials offer excellent flexibility, low thermal conductivity, and high Seebeck coefficients [14,15]. However, their electrical conductivities are often relatively low for effective thermoelectric applications. Enhancing the electrical conductivity of polymer-based thermoelectric generators through doping is possible, yet this method has frequently been shown to be ineffective in significantly improving performance [16]. Consequently, there is a significant need to develop environmentally friendly and cost-effective thermoelectric materials with high thermoelectric figure of merit.

Graphene, graphite, graphene oxide, and carbon nanotube (CNT) are attracting attention for various energy applications due to their widespread availability and environmentally friendly nature [17,18]. Both theoretical and experimental studies have demonstrated that graphene is a promising material for thermoelectric applications due to its tailorable band gap and high charge carrier mobility [19,20,21,22]. Despite their potential, a major challenge with graphene and graphite for thermoelectric use is their high in-plane thermal conductivity [23]. To overcome this, recent studies have explored porous graphene foam structures that are designed to reduce thermal conductivity while maintaining sufficient electrical conductivity and Seebeck coefficient. However, the fabrication methods for creating these foam structures remain complex, posing challenges for scalability and practical application [24,25,26].

In 2014, Lin and colleagues developed a technique to transform commercial polyimide (PI) films into three-dimensional porous laser-induced graphene (LIG) under ambient conditions by irradiating with a mid-infrared (MIR) CO_2_ laser [27]. This irradiation induces lattice vibrations leading to localized high temperatures exceeding 2500 °C. Such intense heat causes the breaking, recombining, and releasing of C-O, C=O, and N-C bonds in the polyimide, producing a porous graphene nanostructure with pentagonal, heptagonal, and hexagonal lattice structures [28]. The remarkable properties of LIG, such as its mechanical flexibility, high porosity, and good electrical conductivity, along with its scalability and ease of production, make it ideal for numerous applications [29,30,31]. These include gas sensors [32], strain sensors [33], supercapacitors [34], triboelectric nanogenerators [35], and flexible heaters [36].

LIG is a promising material for thermoelectric applications due to its high electrical conductivity and tunable work function through laser parameters [37], which potentially influence the Seebeck coefficient. Additionally, LIG exhibits low thermal conductivity owing to its porous structure, a characteristic that is desirable for thermoelectric applications [38]. Despite extensive research on LIG and its applications, there are no studies on the thermoelectric properties of LIG in the existing literature.

In this study, for the first time, the thermoelectric properties of laser-induced graphene were investigated. The influence of laser parameters on the Seebeck coefficient, electrical conductivity, and thermal conductivity of the laser-induced graphene was examined. Additionally, the relationship between thermoelectric properties and the structure of the laser-induced graphene was examined using Raman spectroscopy and SEM. Enhancement of the thermoelectric figure of merit (ZT) through tuning of laser parameters was investigated.

## 2. Materials and Methods

### 2.1. Laser-Induced Graphene Fabrication on Polyimide Film

Laser-induced graphene samples were prepared by first cleaning polyimide Kapton^®^ films, supplied by DuPont (Wilmington, DE, USA), having a thickness of 127 µm, with ethanol and deionized water. Subsequently, the cleaned films were scribed under ambient conditions using a Q-switched pulse fiber laser (Raycus RFL-P50QB, Wuhan Raycus Fiber Laser Technologies Co., Ltd., Wuhan, China) that operates at a wavelength of 1064 nm and has a maximum output power of 50 W. The laser beam was controlled over the surface with a galvanometer scanner equipped with an f-theta lens of 163 mm focal length. The scanning speed, repetition frequency, and laser power were adjustable and controlled via a computer using EzCad2.4.11 software.

In our experiments, we explored the impact of defocusing on the quality of laser-induced graphene (LIG) and determined that positioning the polyimide film 10.5 mm below the laser’s focal point yielded the best results. This finding aligns with the existing literature, which suggests that defocusing can enhance the uniformity and conductivity of LIG by distributing the laser’s energy more evenly across the material surface [39]. With the scanning speed set at 100 mm/s, a line step width of 0.01 mm, and a repetition frequency of 95 kHz, we varied the laser power between 9.75 and 12 W to investigate its effects on the thermoelectric properties of laser-induced graphene.

### 2.2. Characterization

The surface morphology of laser-induced graphene on polyimide substrates was examined using the Scanning Electron Microscope (Thermo Scientific™ Axia ChemiSEM, Waltham, MA, USA). Raman measurements were conducted using Micro-Raman spectroscopy (LabRam 800, Horiba Scientific, Jobin Yvon, France). The excitation was provided by a He-Ne laser with an emission wavelength of 633 nm. Sheet resistance measurements were carried out using the standard four-point probe method with a Keithley DMM6500 (Cleveland, OH, USA). Thermal conductivity (к) values were calculated using Equation (2), wherein the thermal diffusivity (α) and specific heat (C_p_) were measured with a Netzsch LFA 457 MicroFlash system (Netzsch Gerätebau GmbH, Selb, Germany). These measurements were repeated multiple times to ensure accuracy.

к = αρCp (2)

The in-plane thermal diffusivities were measured using an appropriate in-plane holder. To determine the density (ρ) of the samples, the total mass of each specimen was measured using a microbalance scale. The thickness and diameter of the samples were measured with a scanning electron microscope.

### 2.3. Seebeck Coefficient Measurement Setup

**Figure 1:**
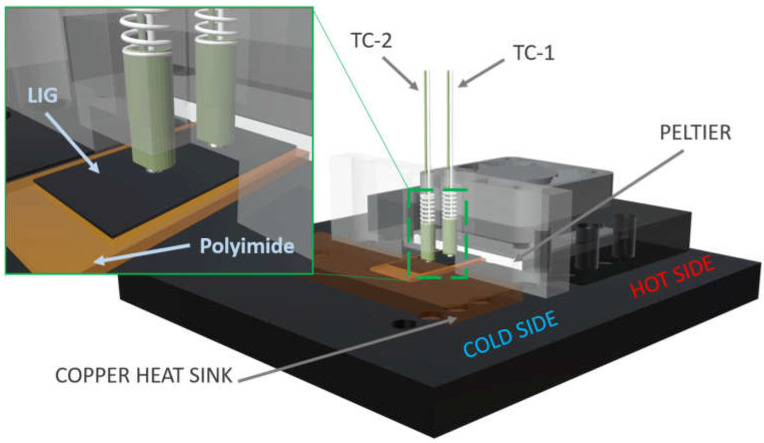
A sketch of home-built Seebeck coefficient setup.

Seebeck coefficient measurements were conducted at room temperature using a custom-built setup, as illustrated in Figure 1. This setup features a Peltier element to maintain the temperature on the hot side and a heat sink to stabilize the temperature on the cold side. To measure the temperature difference (ΔT) across the sample, two K-type thermocouples were positioned on both the hot and cold sides. The minimum four thermovoltage values were recorded in relation to four different ΔT values between 1 °C and 7 °C. Thermovoltage values were measured through the chromel (positive) legs of the thermocouples to ensure that voltage and temperature measurements were taken precisely at the same location on the sample. Thermocouples were spring-loaded to maintain consistent thermal and electrical contact with the sample. A fan was attached to the top side of the Peltier element to maintain constant temperature. Thermovoltage measurements were performed via Keithley multimeter (DMM6500) and temperatures on TC-1 and TC-2 were measured by Cryocon^®^ 24C temperature controller.

In order to accurately calculate the Seebeck coefficient of the sample and eliminate any thermovoltage contributions from the chromel leg of the thermocouple, Equation (3) was applied [40].

S_s_ = −ΔV/ΔT + S_w_ (3)

Here, S_s_ is the Seebeck coefficient of the sample, S_w_ is the Seebeck coefficient of the chromel leg of the K type thermocouple, ΔV is the thermovoltage, and ΔT is the temperature difference. Prior to conducting measurements with the homemade setup, a constantan sample and a nickel foil (both supplied from ULVAC) were measured using a commercial ULVAC ZEM-3 setup (Chigasaki, Japan), and then they were used as reference samples. In order to calibrate the system and calculate S_w_, the constantan reference sample was measured at room temperature. Moreover, to verify the calibration accuracy, the nickel foil was measured subsequent to the calibration process. The Seebeck coefficient of the nickel foil was found to be −16.2 µV/K, with an error rate of 10% when compared to the literature values [41].

## 3. Results

### 3.1. Structural Characterization of Laser-Induced Graphene

**Figure 2:**
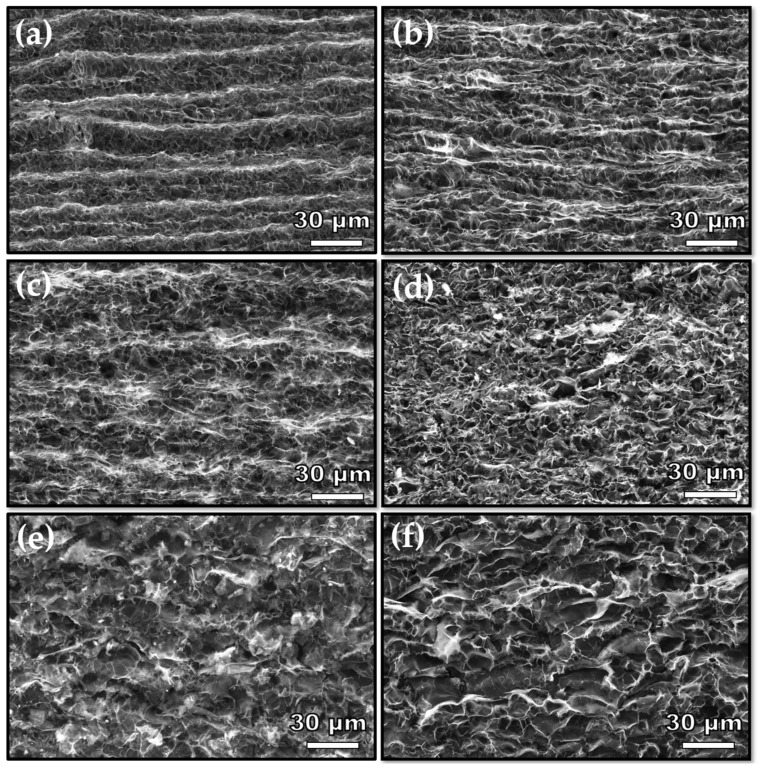
Secondary electron (SE) top-view SEM images of laser-induced graphene samples fabricated with a laser power of (**a**) 9.75 W, (**b**) 10 W, (**c**) 10.5 W, (**d**) 11 W, (**e**) 11.25 W, (**f**) 12 W.

Various laser scribing power trials on polyimide indicated that the threshold value for transforming polyimide into graphene is 9.75 W. Furthermore, the maximum power is 12 W; beyond this value, laser-induced graphene on polyimide begins to delaminate. Figure 2 presents SEM images of six different LIG samples fabricated using varying laser powers. It is evident that with increasing laser power, the laser scribing lines become less distinct. Moreover, increasing the laser power also leads to the formation of larger graphene flakes.

To further investigate the structural alterations in the laser-induced graphene, Raman spectroscopy was employed to analyze the ablated regions. In the Raman spectra, laser-induced graphene (LIG) has three notable peaks: the D peak at approximately 1350 cm^−1^, the G peak at about 1580 cm^−1^, and the 2D peak around 2700 cm^−1^. These peaks are critical for evaluating the structural characteristics of graphene. The D peak indicates the presence of disorder or imperfections. Conversely, the G peak reflects the first-order scattering vibration of sp^2^ hybridized carbon atoms, a sign of graphitization [27]. The 2D peak results from second-order zone-boundary phonons, with its presence and intensity depending on the number of stacked graphene layers. It is widely acknowledged that the intensity ratio of the D to G bands (I_D_/I_G_) serves as a critical measure of structural disorder within graphitic materials. Lower I_D_/I_G_ values indicate greater degrees of graphitization [42].

**Figure 3:**
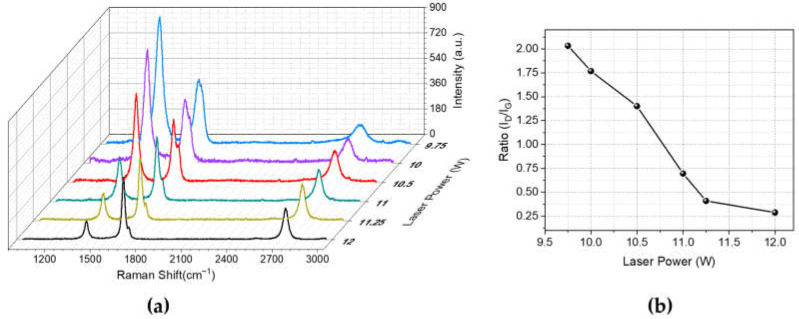
(**a**) Raman spectra of laser-induced graphene on polyimide, produced using different laser power settings; (**b**) I_D_-to-I_G_ intensity ratio as a function of laser power.

In Figure 3, Raman spectroscopy data of laser-induced graphene fabricated with various laser powers is presented. As seen in Figure 3a, the intensity of the D peak located at 1350 cm^−1^ decreases as the laser power increases. Moreover, the 2D peak located at 2700 cm^−1^ showed a slight increase with increasing laser power, indicating that the number of graphene layers decreases. In Figure 3b, laser power versus D peak-to-G peak intensity ratios is given. It shows that higher laser powers cause an increase in graphitization degree and a decrease in structural disorders in graphene.

### 3.2. Thermoelectric Properties of Laser-Induced Graphene on Polyimide Substrate

In order to investigate the thermoelectric performance of laser-induced graphene, multiple samples were consistently fabricated for each laser power setting. Seebeck coefficients (S) and sheet resistances (R_s_) were measured for each sample. In order to calculate the electrical conductivity (σ), the thickness (t) of the LIG layers was measured from the cross-sections of each sample using SEM. Electrical conductivity values were determined using the formula below.

σ = 1/(R_s_ × t) (4)

**Figure 4:**
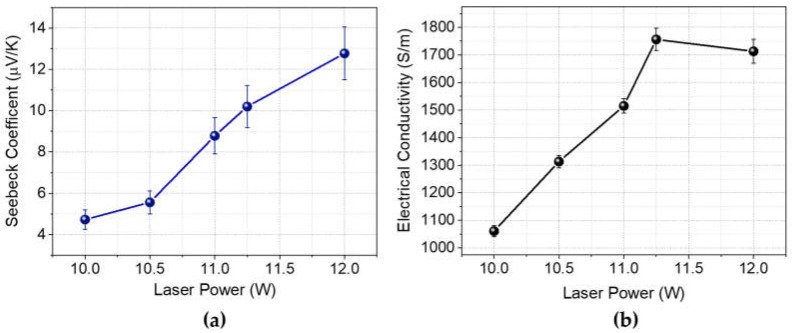
(**a**) Seebeck coefficient, (**b**) electrical conductivity of LIG on polyimide substrate fabricated with various laser powers.

In Figure 4b, electrical conductivity measurements on laser-induced graphene fabricated with various laser powers are given. It can be seen that increasing laser power leads to an increase in electrical conductivity. For the samples scribed with laser powers ranging from 9.75 W to 11 W, a considerable increase in electrical conductivity was observed. However, between 11.25 W and 12 W, no significant changes in electrical conductivity were detected. These observations are consistent with Raman spectroscopy results, suggesting a direct relationship between the degree of graphitization and electrical conductivity. This correlation is possibly due to an increase in the mobility of charge carriers resulting from increased graphitization.

As shown in Figure 4a, the Seebeck coefficient of LIG is positive, indicating p-type doping that is consistent with the existing literature [43]. It is observed that as the laser power increases, the Seebeck coefficient of laser-induced graphene on polyimide also increases. Interestingly, data from Figure 4 indicate a simultaneous increase in both the Seebeck coefficient and electrical conductivity with increasing laser power. The increase in Seebeck coefficient may be attributed to the charge carrier energy filtering effect, which has been widely investigated for carbon allotropes and their nanocomposites [44,45,46]. The charge carrier energy filtering effect suggests that at the interfaces of materials with differing work functions, a potential barrier occurs. This potential barrier scatters low-energy carriers while allowing high-energy carriers to transfer across the interfaces. There are several studies suggesting that introducing defects in graphene results in charge carrier filtering effect [47,48]. To investigate potential defects in laser-induced graphene that cause the energy filtering effect, SEM imaging at higher magnification was conducted.

**Figure 5:**
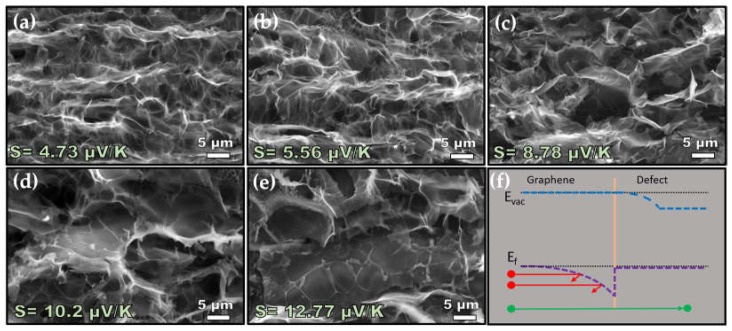
High-magnification SEM images of LIG on polyimide fabricated by (**a**) 10 W, (**b**) 10.5 W, (**c**) 11 W, (**d**) 11.25 W, (**e**) 12 W. (**f**) Energy diagram showing energy filtering at the graphene/defect junction (red sphere is low-energy charge carriers, green sphere is high-energy charge carriers, pink line at the center is the interface.)

Figure 5 presents a high-magnification SEM image of LIG samples scribed with various laser powers. It is observed that as the laser power increases, the size of the graphene flakes also increases. Additionally, certain structures appear on the graphene flakes, becoming more visible particularly after scribing with 11 W of laser power. It should be noted that multiple areas were imaged using SEM, and these structures were observed to be uniformly distributed across the sample. The band diagram in Figure 5f shows the scattering of low-energy holes at the interface between graphene and the defect. Therefore, the increase in graphitization degree leads to increased charge carrier mobility causing higher electrical conductivity. Additionally, as the laser power increases, certain structures form on the graphene flakes, resulting in an energy filtering effect, as shown in Figure 5. This contributes to the increase in the Seebeck coefficient.

**Figure 6:**
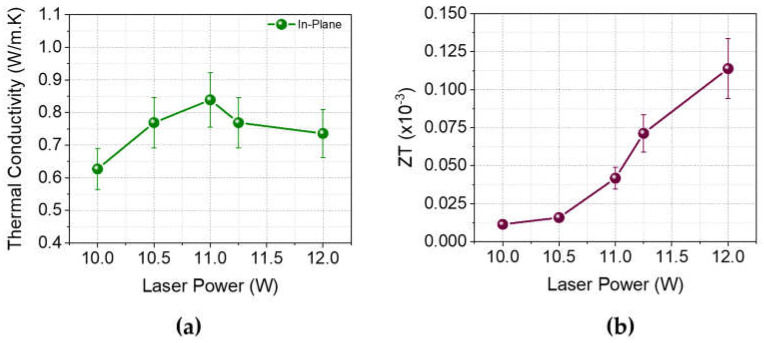
(**a**) In-plane thermal conductivity and (**b**) figure of merit (ZT) in LIG on polyimide substrates fabricated with various laser scribing powers.

To further investigate the thermoelectric performance of LIG samples, in-plane thermal conductivity measurements were performed. Figure 6 shows the in-plane thermal conductivity measurements and calculated thermoelectric figure of merit (ZT) for laser-induced graphene samples fabricated with various laser powers.

As shown in Figure 6a, in-plane thermal conductivity values of LIG on polyimide samples are significantly low. This can be attributed to the porous structure of the LIG, which disrupts heat flow. The measurements reveal that thermal conductivity increases with laser scribing power up to 11 W, after which it begins to decrease. This observation suggests that the structures formed on the laser-induced graphene scatter not only low-energy charge carriers but also serve as scattering centers for phonons. Moreover, the calculated ZT values presented in Figure 6b show a significant increase after the application of 11 W laser scribing power, attributed to the simultaneous increase in electrical conductivity and Seebeck coefficient, and a decrease in thermal conductivity. It was observed that the figure of merit (ZT) of laser-induced graphene (LIG) is enhanced by a factor of 10 when the laser power is increased from 10 W to 12 W.

## 4. Conclusions

In summary, the synthesis and thermoelectric characterization of laser-induced graphene were reported. Increasing the laser power led to enhanced graphitization and the production of larger graphene flakes, which contribute to increased electrical conductivity through greater mobility of charge carriers. As the laser power was raised from 10 W to 12 W, an increase in electrical conductivity from 1061 S/m to 1756 S/m was observed. Also, a simultaneous rise in the Seebeck coefficient with electrical conductivity was detected, ranging from 4.73 µV/K to 12.77 µV/K, which is attributed to the charge carrier energy filtering effect. Furthermore, in-plane thermal conductivity measurements exhibited relatively low values, ranging from 0.6 W/m·K to 0.85 W/m·K, which positively impacts the figure of merit (ZT) values. It was shown that the ZT value of laser-induced graphene can be enhanced nearly tenfold by changing laser parameters. Tuning thermoelectric properties by varying laser parameters paves the way for the further improvement of ZT values. In particular, decorating or doping with other metals or metal oxides may significantly enhance ZT values through band gap and interface engineering.

## Data Availability

Data are contained within the article.
